# A lightweight deep neural network with higher accuracy

**DOI:** 10.1371/journal.pone.0271225

**Published:** 2022-08-02

**Authors:** Liquan Zhao, Leilei Wang, Yanfei Jia, Ying Cui

**Affiliations:** 1 Key Laboratory of Modern Power System Simulation and Control & Renewable Energy Technology, Ministry of Education (Northeast Electric Power University), Jilin City, Jilin, China; 2 College of Electrical and Information Engineering, Beihua University, Jilin City, Jilin, China; 3 Zhuhai Power Supply Bureau, Guangdong Electric Power Corporation, Zhuhai, Guangdong China; Indian Institute of Technology Patna, INDIA

## Abstract

To improve accuracy of the MobileNet network, a new lightweight deep neural network is designed based on the MobileNetV2 network. Firstly, it modifies the network depth of MobileNetV2 to balance the image resolution, network width and depth to keep the gradient stable, which reduces the generation of gradient vanishing or gradient exploding. Secondly, it proposes an improved Bottleneck module by introducing channel attention mechanism. It assigns different weights for different channels according to the degree of relevance between the object features and channels. Therefore, the network can extract more effective features from a complex background. In the end, a new usage strategy of the improved Bottleneck is proposed. It uses the improved Bottleneck module in the second, fourth and fifth stages of MobileNetV2, and uses the original Bottleneck module in other states. Compared with MobileNetV2, MobileNetV3, ShuffleNetV2, GhostNet and HBONetmethods, the proposed method has the highest classification accuracy on the ImageNet-1K dataset, CIFAR-10 and CIFAR-100. Compared with YOLOV4-Lite methods based on these lightweight network networks, YOLOV4-Lite based on our proposed network also has the highest detection accuracy on the PASCAL VOC07+12 dataset.

## Introduction

With the development of graphic processor unit technology, deep convolutional neural networks have aroused more and more attentions. To improve accuracy, more and more deep convolutional neural networks with complexity constructs are designed and applied in image classification [1], fault diagnosis [2] and image Dehazing [3], etc. They have a complex network structure and a larger number of network parameters. Therefore, they require to be deployed on a server with powerful computing power. However, mobile devices and embedded devices (autonomous driving devices, augmented reality devices and other smart devices) have limited computing power and limited memory, and cannot be deployed on for the common deep convolutional neural network. These limit the application of deep learning in real-time detection. To solve the problem, lightweight deep convolutional neural networks are proposed. Compared with the common deep convolutional neural network, they have a simpler network structure and can be deployed on devices that have limited computing power and memory (such as telephone mobile, Jeston nano and Raspberry Pi) [4–6]. They are also have been widely used in vehicle detection [7], pedestrian detection [8], bus passenger object detection [9], agricultural detection [10], human abnormal behavior detection [11], etc.

The lightweight deep convolutional neural networks can be designed by compressing the normal convolutional neural networks by quantization, pruning and knowledge distillation methods [12–14]. These methods can reduce the complexity and computation cost of the network. Another kind of method is to directly design lightweight convolutional neural networks. Such as ShuffleNet series methods, SqueezeNet series methods and MobileNet series methods. MobileNet is one of the typical lightweight deep convolutional neural networks. It is designed for mobile and embedded vision applications. The MobileNets [15] constructed lightweight deep neural networks by using depthwise separable convolution instead of the traditional convolution to reduce parameters. To improve the ability to extract features, MobileNetV2 designed an Bottleneck module by adding the point-wise convolution layer [16]. To improve detection speed, MobileNetV3 improved computationally-expensive layers and introduced the hard swish nonlinearity on the basis of MobileNet to reduce computation complexity [17].

To further improve the accuracy of lightweight convolutional neural network, we propose a lightweight convolutional neural network based on MobileNetV2. The network layer depth directly affects the classification accuracy with the same network width and resolution of the input image (224 × 224). In our simulation analysis, the original network depth of MobileNetV2 is not the optimal network depth. Therefore, we modify the network depth by reducing the number of Bottleneck modules that are used to extract features from the 14× 14 feature map to improve the accuracy according to our simulation research. The channel number of the deep convolution layer is the most in the Bottleneck module, and the channels are independent of each other. The different channels have different contributions to the classification task. The more important feature the channel contains, the larger the weight of the channel is. Therefore, we introduce the channel attention module into the Bottleneck module to make the feature extraction network pay more attention to the channels that contain more important feature. This can make the feature extraction network extract more effective features. The weight of the improved Bottleneck module is in the range 0 to 1, and the output of the feature is the product of feature and weight. If we construct the whole network by continually stacking the improved Bottleneck module, it will reduce the value of the extracted feature and affects accuracy. To avoid the problem, we alternately use the improved Bottleneck module and original Bottleneck module to construct the whole network.

The contributions of our proposed method are summarized as follows:

1. We reduce the number of Bottleneck modules that are used to construct MobileNet to balance the network layer width and depth. This also can reduce the generation of gradient vanishing and gradient exploding.
2. We improve the Bottleneck module by introducing the channel attention mechanism. This makes the channels that contain more important features related to the classification task obtain a larger weight. It is useful for extracting more effective features.
3. We also propose a new strategy to construct a lightweight convolutional neural network with higher accuracy by using the improved Bottleneck module and original Bottleneck module. Compared with the current lightweight networks, the proposed lightweight network has better performance in accuracy.

## Literature review

The typical lightweight deep convolutional neural network includes SqueezeNet, ShuffleNet and MobileNet. Iandola et al. [18] proposed a fire module and stacked the fire modules to construct SqueezeNet. The fire module was composed of a squeeze layer and an expand layer. The squeeze layer was mainly composed of 1×1filter. The main function of this layer was to reduce the number of input channels of the expand layer. The 1×1 filter replaced part of the 3×3 filter in the expand layer. It can achieve the same classification accuracy as AlexNet, but the parameter amount is 1/50 of AlexNet. Gholami et al. [19] proposed SqueezeNext on the basis of SqueezeNet. It designed two-stage bottleneck modules to reduce parameters and lower rank filters to reduce redundancy of convolution kernel. Its neural network performance is able to achieve AlexNets top-5 performance. Zhang et al. [20] proposed ShuffleNet. It used pointwise group convolution instead of 1×1 convolution operation to reduce the complexity of convolution operation. In order to overcome the side effects of group convolution, it also proposed a channel shuffle operation to improve feature information flow between channels. Ma et al. [21] proposed ShuffleNetV2. They found that the extensive use of group convolution will increase the memory access cost (MAC)and reduce the speed of the model. In order to solve this problem, they introduced the channel split operation on the basis of ShuffleNet to respectively divide the input features into two parts as the input of the identity mapping branch and the feature extraction branch. At the same time, the pointwise group convolution in the feature extraction branch is replaced by the traditional convolution with the convolution kernel size of 1×1 and the concat operation is used instead of the add operation in ShuffleNet to connect the outputs of the two branches. It reduced the complexity of the network and improved classification speed. In order to reduce the parameters and complexity of the network. Han et al. proposed a novel lightweight network that is GhostNet [22]. The network was mainly composed of 1×1 convolution and 3×3 depthwise convolution. In order to use a few convolution kernels without reducing the number of network feature maps, it firstly used a small number of 3×3 depthwise convolutions to generate a part of the feature maps. Secondly, it used linear operation to generate more feature maps. Finally, the 1×1 convolution is used to fuse these feature maps.

Howard et al. [15] proposed MobileNets based on depthwise separable convolution that consisted of pointwise convolution and depthwise convolution. In the depthwise convolution, each convolution kernel only convolves with the feature map of one channel. In the common convolution, one convolution kernel convolves with the feature maps of all channels. Compared with the normal convolution, depthwise convolution can extremely reduce the number of network calculations and parameters. Point convolution is to fuse the feature maps generated by depthwise convolution to promote the exchange of information between channels. Besides, it also introduced two simple global hyper-parameters to balance the accuracy and network complexity. Howard et al. [16] proposed MobileNetV2 based on MobileNets. Compared with MobileNets, it increased the Bottleneck module and linear bottleneck module. The Bottleneck module is composed of three convolutional layers, the first and last layers are pointwise convolution, and the middle layer is depthwise convolution with a 3×3convolution kernel. To enable the 3×3 depthwise convolutional layer to learn more features and improve the accuracy of the network, the number of output channels in the first layer is six times the number of input channels. To avoid adding more network parameters and calculations, the number of output channels in the last layer of the module is less than the number of input channels. The last layer is pointwise convolution without the ReLU activation function. Howard et al. [17] proposed MobileNetV3 based on Auto ML technology. This structure not only includes depthwise separable convolutions, Bottlenecks, and linear bottlenecks, but also SE models and the new activation functions h-swish. Therefore, this network can achieve better performance with fewer FLOPs.

Wang et al. [23] introduced the dense blocks proposed in DensNets into the MobileNet model to further reduce the number of network parameters and improve the classification accuracy. It used the convolutional layers with the same size of the output feature map as dense blocks and used dense connections in the dense blocks to extract richer features. This network structure can repeatedly use the output feature map generated by the previous convolution layer in dense blocks to reduce the convolution cores. Li et al. [24] combined the SSD network as a meta-structure with MobileNets and proposed a MobileNet-SSD network. It optimized the SSD structure without sacrificing accuracy. Pan et al. [25] proposed a TL-MobileNet model to quickly and effectively monitor the welding defects to ensure the quality of the welded structure. The MobileNets with FC-128 fully connected layer and Softmax classifier were added as the feature extractor of welding defects, and the model was optimized using Drop Block technology and global average pooling.

## The proposed lightweight deep neural network

### Proposed network depth

In the common researches, they increase the network depth to improve accuracy. However, with the increase of network depth, it increases the generation of gradient vanishing and gradient exploding. This will directly affect network accuracy. Besides, Tang and Le also point that balancing network width, depth and image resolution can make the network have better classification accuracy [26]. Increasing network width will increase the number of channels, which makes the network extract more features. On the contrary, reducing network width also will affect feature extraction. To avoid increasing network complexity, we keep the network width unchanged in the MobileNet network. Besides, the image resolution often used in model training is 224×224, and the network with this size of the image resolution has good generalization ability in the MobileNet network. Therefore, we also keep the image resolution unchanged. If we increase the depth of the network, the complexity of the network will also be increased and gradient vanishing and gradient exploding may be generated in the MobileNet network. Therefore, we consider reducing the depth of the MobileNet network to improve accuracy.

In the MobileNetV2 network, the network can be divided into five stages according to the size of the input feature map, and each stage is composed of one or several stacks of Bottleneck modules with the same structure. When the initial input image size of the network is 224×224, the sizes of the input feature map in the feature extraction stage are 112×112, 56×56, 28×28, 14×14, and 7×7 in sequence. According to the size of the input feature map in each stage, the network can be divided into 5 stages, and the numbers of Bottleneck modules in each stage are 3, 3, 4, 6, and 1, respectively. The shallow feature extraction networks are mainly to extract spatial information such as color and edge. The deep feature extraction networks are mainly to extract features from the feature map that is the output of the shallow feature extraction network and fuse the different features, to obtain the deep semantic information. If the depth of the shallow network is reduced, it will cause the loss of spatial information, and directly affect the deep semantic information extraction. The fifth stage is the last layer with only one Bottleneck module. The fourth stage has the most Bottleneck modules and also is the deep feature extraction network. Therefore, we try to reduce the number of Bottleneck modules in the fifth stage to improve network accuracy.

We set the numbers of the Bottleneck module as 4, 5 and 6 in the fourth stage in the MobileNetV2 network, respectively. We test the MobileNetV2 network with different numbers of the Bottleneck module in the fourth stage on the ImageNet-100 dataset, CIFAR-100 dataset and CIFAR-10 dataset, respectively. The ImageNet-100 dataset contains 129,395 training images and 5000 test images with 100 categories. CIFAR-100 dataset has 100 categories, and each category contains 600 images. The training set of CIFAR-100 dataset contains 50000 images that is obtained by selecting 500 images from each category. The remaining 10000 images are used as the training set. CIFAR-10 dataset has 10 categories, and each category contains 6000 images. The training set of CIFAR-10 dataset contains 50000 images that is obtained by selecting 5000 images from each category. The remaining 10000 images are used as the training set. Table 1 shows the Top-1accuracy, Parameters, and FLOPs for different numbers of the Bottleneck module in the fourth stage on the ImageNet-100 dataset. The MobileNetV2(6), MobileNetV2(5), and MobileNetV2(4) are MobileNetV2 networks based on 6, 5, and 4 Bottleneck modules in the fourth stage, respectively. In Table 1, the TOP-1 accuracy of MobileNetV2(6) is the lowest. Compared with MobileNetV2 (6), the TOP-1 accuracy of MobileNetV2(5) is increased by 0.66% and the MobileNetV2(4) is increased by 0.35%. The MobileNetV2(5) has the highest TOP-1 accuracy. Table 2 shows the results on CIFAR-100. In Table 2, Compared with MobileNetV2(6), the TOP-1 accuracy of MobileNetV2(5) is increased by 0.52% and the MobileNetV2(4) is decreased by 0.28%. The MobileNetV2(5) still has the highest TOP-1 accuracy. Table 3 shows the results on CIFAR-10. In Table 3, Compared with MobileNetV2(6), the TOP-1 accuracy of MobileNetV2(5) is increased by 0.55% and the MobileNetV2(4) is decreased by 0.36%. The MobileNetV2(5) still has the highest TOP-1 accuracy. From Tables 1–3, we can see that parameters and FLOPs increase with the increase of Bottleneck module number. Although the MobileNetV2(4) network has higher accuracy than MobileNetV2(6) on ImageNet-100 and lower accuracy on CIFAR-100 and CIFAR-100 datasets, the MobileNetV2(5) network still has the highest accuracy on ImageNet-100, CIFAR-100 and CIFAR-10 datasets. Therefore, we reduce the number of Bottleneck modules from 6 to 5 in the fourth stage to obtain better accuracy performance.

**Table 1:** Results on IMAGENET-100.

*Model*	TOP-1(%)	Parameters	FLOPs
*MobileNetV*2(6)(*baseline*)	77.86	3.50M	314.19M
*MobileNetV*2(5)	78.52(+0.66)	3.18M	298.51M
*MobileNetV*2(4)	78.21(+0.35)	2.86M	282.83M

**Table 2:** Results on CIFAR-100.

*Model*	TOP-1(%)	Parameters	FLOPs
*MobileNetV*2(6)(*baseline*)	77.79	3.50M	314.19M
*MobileNetV*2(5)	78.31(+0.52)	3.18M	298.51M
*MobileNetV*2(4)	77.51(-0.28)	2.86M	282.83M

**Table 3:** Results on CIFAR-10.

*Model*	TOP-1(%)	Parameters	FLOPs
*MobileNetV*2(6)(*baseline*)	92.34	3.50M	314.19M
*MobileNetV*2(5)	92.89(+0.55)	3.18M	298.51M
*MobileNetV*2(4)	91.89(-0.36)	2.86M	282.83M

### Network structure

#### Network structure of original MobileNetV2

**Fig 1:**
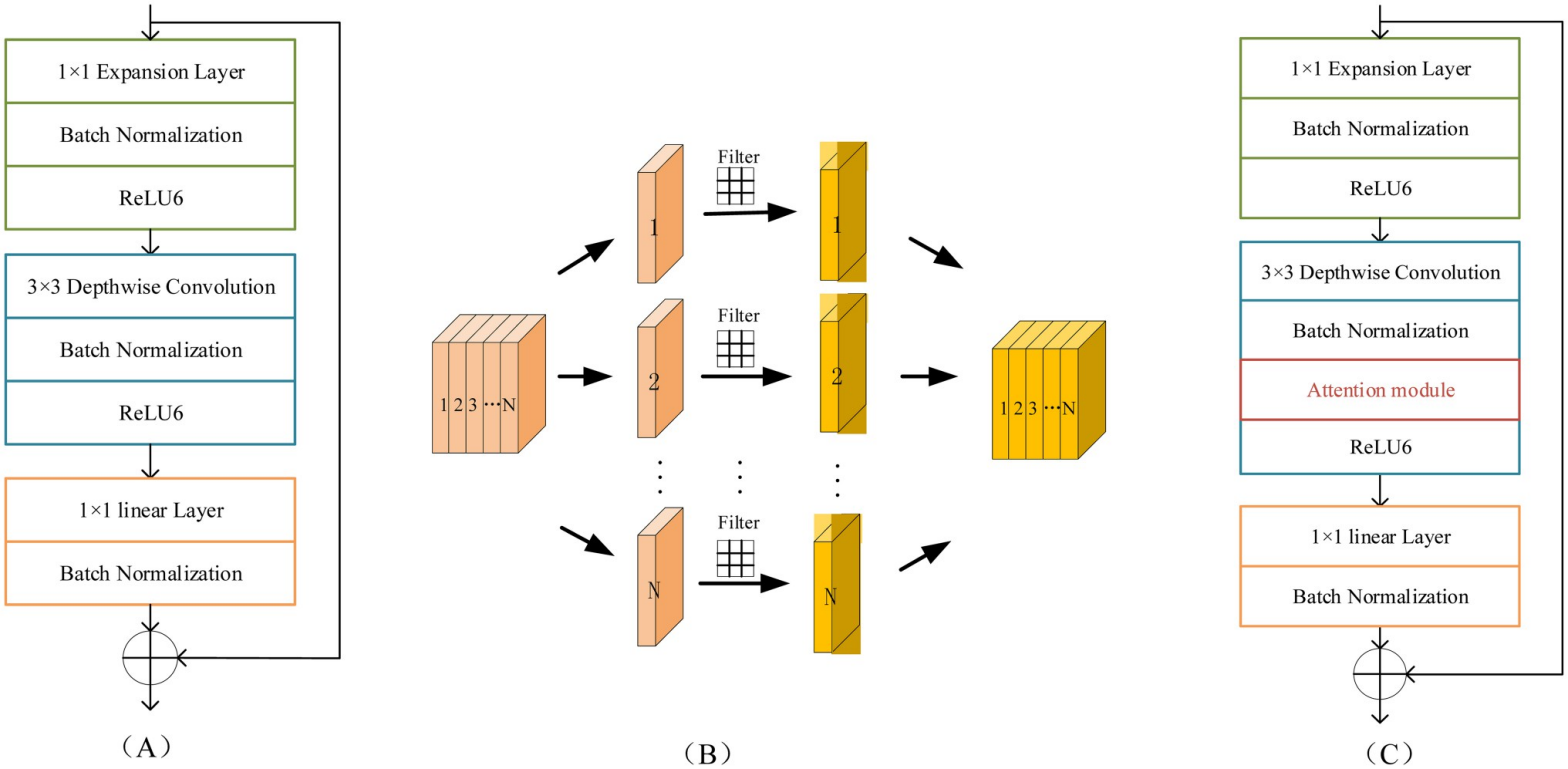
The original Bottleneck module and the improved Bottleneck module. A: original Bottleneck module. B:depthwise convolution. C: improved Bottleneck module.

The original MobileNetV2 network is mainly composed of many Bottleneck modules. The Bottleneck module is shown in Fig 1(A). It consists of three parts. The first part is composed of a 1×1 convolutional layer, BN layer, and ReLU6 activation function. In order to make the model have a richer feature output, the number of output channels is six times that of input channels in the first part. The second part is composed of a 3×3 depthwise convolution layer, a BN layer, and a ReLU6 activation function. The number of output channels of this layer is the same as the number of input channels. The final part is composed of a 1×1 convolutional layer and a BN layer. The main function of the final part is to reduce the dimension of the channel and complexity of the module. The depthwise convolution used in the Bottleneck module shown in Fig 1(A) is shown in Fig 1(B). Each channel corresponds to only one convolution kernel, and the channels are independent of each other and contain different feature information.

The detailed structure of the original lightweight convolutional neural network is shown in Table 3. The In_C and Out_C denote the number of input channels and output channels, respectively. n denotes the number of Bottleneck block. S denotes the convolution step of the depthwise convolution layer in the last Bottleneck module. t represents the channel expansion multiple. The Bottleneck is the original Bottleneck module that shown in Fig 1(A).

#### Network structure of proposed MobileNetV2

To enable the inverted residual module to obtain more effective feature information related to the classification task in a complex background, we introduce the channel attention into the network. It can assign different weights to different channels according to the relationship between the channel and key information of the image. The channel contains more important features related to the classification task, the weight of the channel will be larger. This makes the network pay more attention to the channel that contains the important feature and reduces the influence of useless information.

**Fig 2:**
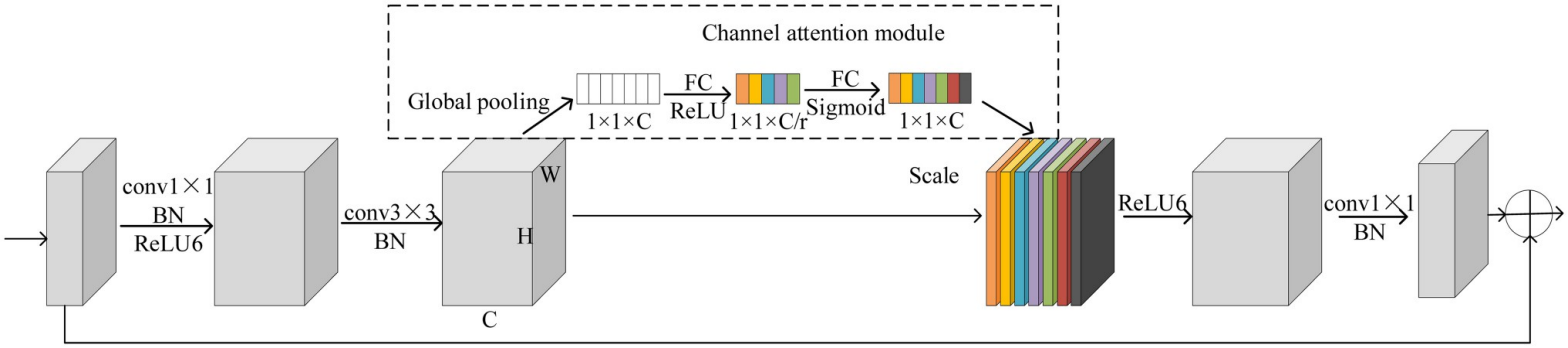
The detail network structure of improved Bottleneck module with the attention mechanism.

The improved inverted residual module and is shown in Fig 1C. We add the channel attention module between the BN layer and the ReLU6 activation function in the second part of the inverted residual module. Fig 2 shows the detailed network structure of the improved inverted residual module. The gray rectangle expresses the feature map that is the output of previous layer network. The first layer is a 1×1 convolutional layer connected with a BN layer and ReLU6 activation function. The channel number of output feature map is six times that of input feature map to make the network have a richer feature output. The second layer is a 3×3 depthwise convolution layer connected with a BN layer. The structure of depthwise convolution is shown in Fig 1B. The number of input feature is the same with the number of channels for depthwise convolution layer. The channel number of output feature map is the same with that of input feature map for the second layer. To make the channel that contains more important feature information receive more attention, we introduce the channel attention module into the network. In the inverted residual module, it has the largest number of channels and a larger receptive field, so the output feature will contain more information. Therefore, we insert the attention module after the 3×3 depthwise convolution layer. The input feature map of channel attention module is the output feature map of the second layer (3×3 depthwise convolution layer). In the channel attention module that is shown in Fig 2, the global average pooling operation is used to compress the feature map with the size H×W in each channel into 1×1. The output of the global average pooling operation is used to represent the global information of the corresponding channel. The output of the global average pooling for cth channel can be expressed as following:

$$\begin{array}{c} x_{c}=\frac{1}{H\times W}\sum_{i=1}^{H}\sum_{j=1}^{H}y_{c}\left(i,j\right) \end{array}$$ (1)

where *y*_*c*_ is the output feature map of cth channel for second layer. The global average pooling operation is connected with two fully connected layers that are used to learn features for each channel. To reduce the complexity of the network and strengthen the generalization ability, the dimension of the first fully connected layer is reduced to c/r (r is a hyperparameter, c is the number of the channel). The ReLU activation function is used behind the first fully connected layer, and the sigmoid activation function is used for the last fully connected layer. It output of channel attention module is

$$\begin{array}{c} z=\sigma (w_{2}\delta \left(w_{1}x\right)) \end{array}$$ (2)

where *w*_1_ and *δ* are the weight and ReLU activation function of the first fully connected layer, respectively. *w*_2_ and *σ* are the weight and sigmoid activation function of the second fully connected layer, respectively. Finally, through a scale operation, the normalized relationship weight is weighted to the characteristics of each channel. The output feature map is

$$\begin{array}{c} o_{c}=z_{c}y_{c} \end{array}$$ (3)

where *y*_*c*_ is the output feature map of cth channel for second layer. *z*_*c*_ is the weight of cth channel. The more relevant the features contained in the channel are to the classification task, the larger the channel weight is. This can make the channel that contains more important feature information receive more attention. *o*_*c*_ is connected with ReLU6 activation function, 1×1 convolutional layer and a BN layer.

In the improved Bottleneck block, the channel weight is distributed in [0, 1]. The output feature is the product of feature and channel weight. If we construct the MobileNet network by directly stacking the improved Bottleneck blocks, the channel weight will be multiplied by the feature matrix points continuously. This will reduce the value of features and affect the change of gradient, especially in the case of a deep network, which will bring great difficulties to training. Besides, it may alter the attribute of identity mapping in the original network module. To alleviate the problem, we construct a network by alternately using improved Bottleneck block and original Bottleneck block in different stages. The deeper network layer contains more feature information and stronger the generalization. Therefore, we use the improved Bottleneck block in the second stage and the fourth stage. The original fifth stage has only one Bottleneck block, so we also use the improved Bottleneck block in the final stage.

The detailed network structure of the improved MobileNetV2 is shown in Table 5. The In_C and Out_C denote the number of input channels and output channels, respectively. n denotes the number of Bottleneck block. S denotes the convolution step of the depthwise convolution layer in the last Bottleneck module. t represents the channel expansion multiple. The Bottleneck is the original Bottleneck module. The IBottleneck is our improved Bottleneck module that is shown in Fig 1(C). The first layer of the network is composed of a standard 3×3 convolution. According to the size of the input feature map in each stage, the network can be divided into 5 stages. For the first stage, the size of the input map is 112×112. It consists of three original bottleneck residual blocks. The number of channels for the first bottleneck residual block is 32, and the number of channels for the other two bottlenecks residual block is 16. For the second stage, the size of the input map is 56×56. It consists of three improved bottleneck residual blocks. For the third stage, the size of the input map is 28×28. It consists of four original bottleneck residual blocks. For the fourth stage, the size of the input map is 14×14. It consists of five improved bottleneck residual blocks. The number of channels is 64 for the first three improved bottlenecks residual and the number of channels for the other improved bottleneck residual is 96 in the fourth stage. In the final stage, it consists of an improved bottleneck residual block, two standard 1×1 convolutions and an average polling.

Compared with the network structure of original MobileNetV2 shown in Table 4, there are two main differences between the improved MobileNetV2 and original MobileNetV2. The two differences are marked in bold font in Table 5 and summarized as follows:

1. For the input map with size 14×14, the numbers of channel expansion multiple are 6 and 5 for original MobileNetV2 and improved MobileNetV2, respectively.
2. The original MobileNetV2 use original inverted residual module (Bottleneck module) to construct the whole network. We use our improved inverted residual module (IBottleneck module) and original inverted residual module (Bottleneck module) to construct the whole network.

**Table 4:** The network structure of improved MobileNetV2.

*Inputsize*	In_C	Operator	n	t	out_c	s
224×224	3	conv2d	1	−	32	2
112×112	32	Bottleneck	1	1	16	1
112×112	16	Bottleneck	2	6	24	2
56×56	24	Bottleneck	3	6	32	2
28×28	32	Bottleneck	4	6	64	2
14×14	64	Bottleneck	3	6	96	1
14×14	96	Bottleneck	3	6	160	2
7×7	160	Bottleneck	1	6	320	1
7×7	320	Conv2d1×1	1	−	1280	1
7×7	1280	Avgpool 7×7	1	−	−	−
1×1	1280	Conv2d1×1	1	−	k	−

**Table 5:** The network structure of improved MobileNetV2.

*Inputsize*	In_C	Operator	n	t	out_c	s
224×224	3	conv2d	1	−	32	2
112×112	32	Bottleneck	1	1	16	1
112×112	16	Bottleneck	2	6	24	2
56×56	24	**IBottleneck**	3	6	32	2
28×28	32	Bottleneck	4	6	64	2
14×14	64	**IBottleneck**	**3**	6	96	1
14×14	96	**IBottleneck**	2	6	160	2
7×7	160	**IBottleneck**	1	6	320	1
7×7	320	Conv2d1×1	1	−	1280	1
7×7	1280	Avgpool 7×7	1	−	−	−
1×1	1280	Conv2d1×1	1	−	k	−

## Simulation and discussion

For the ImageNet-1K datatset with 1000 categories, 1281167 training images and 100000 test images, different methods use the same mini-batch size that is 256. Cosine annealing learning rate is used in the training process [27]. The initial learning rate is 0.05. The total number of epochs is 200. The momentum of the SGD optimizer is 0.9, and the weight decay is 0.00004. For the CIFAR100 and CIFAR10 datasets, the mini-batch size is 128. Cosine annealing learning rate is also used in the training process. The initial learning rate is 0.1. The total number of epochs is 100. The momentum of the SGD optimizer is 0.9, and the weight decay is 0.0001. For the PVOC07+12 dataset, the mini-batch size is 16. The initial learning rate is 0.0001. The number of epochs is 100. The momentum of the SGD optimizer is 0.9, and the weight decay is 0.0005. The input image resolution is 416×416. The VOC07+12 dataset is composed of the training set of the PASCAL VOC2007 and VOC2012 datasets. It contains 21503 images with 20 categories. The training dataset contains 16551 images with 40058 objects. The test dataset contains 4952 images with 12032 objects. The experimental environment is that the operating system is Ubuntu18.04. The CPU is Intel Xeon E5-2678 v3 at 2.5 GHz. The GPU is NVIDIA GeForce GTX 1080Ti. The memory is 64G. The learning framework is PyTorch.

### Ablation study

The network structure of improved MobileNetV2 is shown in Table 5. It consists of original Bottleneck, improved Bottleneck, convolution layers and pooling layer. We introduce the attention module into improved Bottleneck. To analyze the influence of attention module on the network, the improved MobileNetV2 that consists of improved Bottleneck without attention module is named MobileNetV2(5). The improved MobileNetV2 that consists of improved Bottleneck by adding attention module between the BN layer and ReLU activation function is named MobileNetV2 (5)_M. The improved MobileNetV2 that consists of improved Bottleneck by adding attention module in the end of Bottleneck module is named MobileNetV2 (5)_E. The classification results on ImageNet-100 for original MobileNetV2, MobileNetV2 (5), MobileNetV2 (5)_M, and MobileNetV2 (5)_E are shown in Table 6. The TOP-1 accuracies of MobileNetV2 (5)_M, MobileNetV2 (5)_E, MobileNetV2(5) and MobileNetV2 are 80.01%, 79.34%, 78.52% and 77.86%, respectively. Compared with the MobileNetV2, the TOP-1 accuracies of MobileNetV2 (5)_M, MobileNetV2 (5)_E and MobileNetV2 (5) are increased by 2.15%, 1.48% and 0.66%, respectively. It shows that adding the attention module can improve the accuracy of MobileNetV2(5), and adding the attention module in the middle of Bottleneck module is better than in the end of the Bottleneck module.

**Table 6:** Results for different position of attention module in bottleneck.

*AttentionModule*	TOP-1(%)	Parameters	FLOPs
*MobileNetV*2(*baseline*)	77.86	3.50M	314.19M
*MobileNetV*2(5)	78.52(+0.66)	3.18M	298.51M
MobileNetV2(5)_E	79.34(+1.48)	3.28M	312.25M
MobileNetV2(5)_M	80.01(+2.15)	3.67M	323.70M

Based on the analysis of Table 6, adding the attention module in the middle of Bottleneck module is optimal. Therefore, we use the proposed Bottleneck module by adding attention module between the BN layer and ReLU activation function to improve MobileNetV2. Our improved MonileNetV2 network has five different sizes of input feature map that are 112×112, 56×56, 28×28, 14×14, and 7×7, respectively. According to the sizes of the input feature map, it can be divided into five stages. The numbers of Bottlenecks are 3, 3, 4, 5, and 1 for the five stages in improved MonbileNetV2 network, respectively. Although we have proposed that we use the improved Bottleneck in the second, fourth, and fifth stages of the MonileNetV2 network shown in Table 5, to analyze the influence of position of improved Bottleneck on the network, we also propose to use the Improve Bottleneck in different stages to construct improved MonileNetV2. Therefore, we construct three improved MonileNetV2 networks that are M0, M1, and M2 based on improved Bottleneck. For the M0 network, the original Bottleneck module is used as the Bottleneck module in all stages. For the M1 network, improved Bottlenecks is used as Bottlenecks module in all stages. The M2 network is our finally proposed method. It uses the original Bottleneck in the first and third stages, and the improved Bottleneck in the second, fourth and fifth stages. The results for different position of improved bottleneck in network are shown in Table 7. The TOP-1 accuracies of M0, M1 and M2 are 78.52, 79.73 and 80.01, respectively. Compared with M0, the accuracies of M1 and M2 are increased by 1.21% and 1.49%, respectively. The M2 network has the higher TOP-1 accuracy than others. This shows that our proposed usage strategy of improved Bottleneck and Bottleneck is optimal.

**Table 7:** Results for different position of improved bottleneck in network.

	Stage 1	Stage 2	Stage 3	Stage 4	Stage 5	Top-1
MO	Original Bottleneck	Original Bottleneck	Original Bottleneck	Original Bottleneck	Original Bottleneck	78.52
M1	Improved Bottleneck	Improved Bottleneck	Improved Bottleneck	Improved Bottleneck	Improved Bottleneck	79.73
M2	Original Bottleneck	Improved Bottleneck	Original Bottleneck	Improved Bottleneck	Improved Bottleneck	80.01

### Comparison of different lightweight networks on classification

We compare our proposed network with different lightweight networks that are ShuffleNetV2 [21], GhostNet [22], HBONet [28], MobileNetV3 [17], MobileNetV2 [16] on ImageNet-1K, CIFAR100 and CIFAR10 datasets, respectively. In order to maintain the balance between the number of parameters and FLOPs, the ShuffleNetV2 network width is set to 1.5× and the other network widths are all 1.0×. The TOP-1 accuracies, parameters, and FLOPs(Floating-point Operations per Second) on ImageNet-1K dataset for all networks are shown in Table 8. In the aspect of Top-1 accuracy, our proposed network has the highest value(75.04%), followed by HBONet(73.53%) and MobileNetV3(73.27%). In the aspect of Parameters, the MobileNetV2(baseline network) has the smallest value(3.50M), followed by ShuffleNetV2(3.51M)and our proposed network. In the aspect of FLOPs, the GhostNet has the smallest value(148.79M), followed by MobileNetV2(314.19M), HBONet(316.07M) and our proposed network(323.70M). Compared with MobileNetV2, our proposed method has higher TOP-1 accuracy, more parameters and FLOPs. Compared with MobileNetV3, our proposed method still has higher TOP-1 accuracy, fewer parameters and more FLOPs. Compared with HBONet, our proposed method has higher TOP-1 accuracy, fewer parameters and more FLOPs. The TOP-1 accuracies for different networks on CIFAR100 and CIFAR10 datasets are shown in Table 9, respectively. On the CIFAR-10 and CIFAR-100 dataset, compared with MobileNetV2(baseline network), the Top-1 accuracies are increased by 2.37% and 2.18%, respectively. For the two datasets(the CIFAR10 and CIFAR100 datasets), the proposed method still has the highest TOP-1 accuracy, followed by GhostNet and HBONet. On the whole, our proposed network has the best performance in classification accuracy than other networks. Although it has more parameters and FLOPs, it is still a lightweight convolutional neural network.

**Table 8:** TOP-1 accuracies on on ImageNet-1K dataset.

Lightweight network	TOP-1(%)	Parameters	FLOPs
ShuffleNetV2	71.66	3.51 M	304.51 M
GhostNet	72.22	5.18 M	148.79 M
HBONet	73.53	4.56 M	316.07 M
MobileNetV3	73.27	5.48 M	229.65 M
MobileNetV2(baseline)	72.07	3.50 M	314.19 M
Our proposed network	75.04	3.67 M	323.70 M

**Table 9:** TOP-1 accuracies on CIFAR100 and CIFAR10 datasets.

Lightweight network	CIFAR-10	CIFAR-100
ShuffleNetV2	91.49	77.16
GhostNet	93.38	78.21
HBONet	92.96	77.94
MobileNetV3	91.82	77.39
MobileNetV2(baseline)	92.34	77.79
Our proposed network	94.71	79.97

### Comparison of different lightweight networks on object detection

**Fig 3:**
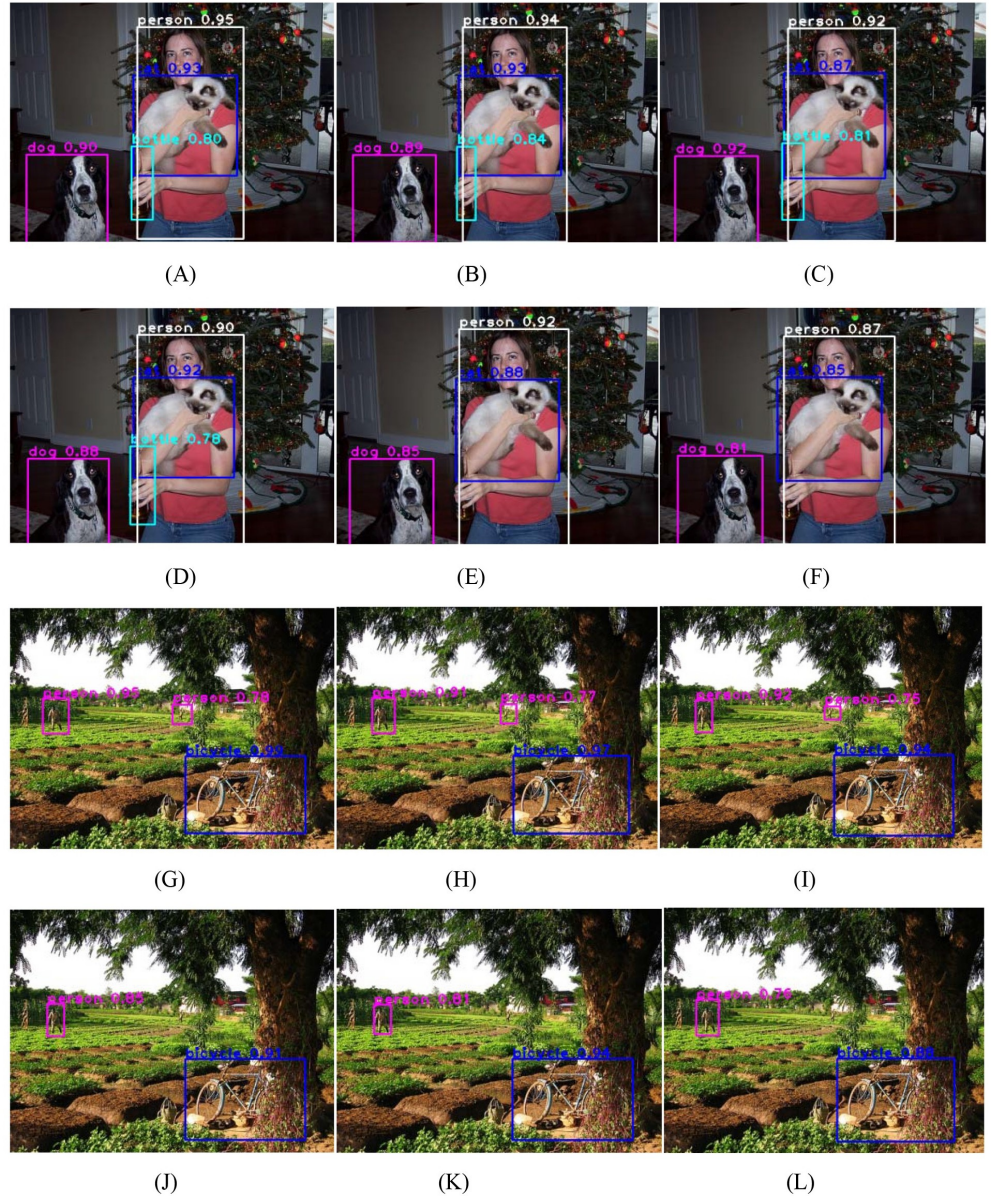
Object detection based on different lightweight networks.

YOLOv4-Lite is an improved YOLOv4 [29]. YOLOV4-Lite has high detection accuracy and fast training speed. We use lightweight convolutional neural networks as the backbone of YOLOv4-Lite to reduce the complexity of YOLOv4-Lite, and test the YOLOv4-Lite based on different lightweight convolutional neural networks on the PASCAL VOC07+12 dataset. We select two images containing objects that are difficult to detect from test dataset to test different networks. The object detection based on different lightweight networks are shown in Fig 3. The Fig 3(A)–3(F) are the detection results based on YOLOV4-Lite + our proposed network, YOLOV4-Lite + MobileNetV3,YOLOV4-Lite +GhostNet, YOLOV4-Lite + MobileNetV2, YOLOV4 +HBONet and YOLOV4-Lite+ShuffleNetV2, respectively. The Fig 3(G)–3(L) are the detection results based on YOLOV4-Lite + our proposed network, YOLOV4-Lite + MobileNetV3,YOLOV4-Lite +GhostNet, YOLOV4-Lite + MobileNetV2 YOLOV4 +HBONet and YOLOV4-Lite+ShuffleNetV2, respectively. On the Fig 3(A)–3(F), the YOLOV4-Lite + our proposed network, YOLOV4-Lite + MobileNetV3,YOLOV4-Lite +GhostNet and YOLOV4-Lite + MobileNetV2 detect all four objects, while the YOLOV4 +HBONet and YOLOV4-Lite+ShuffleNetV2 only detect three objects. The color of bottle is close to background, so it is difficult to detect. On the Fig 3(G)–3(L), the YOLOV4-Lite + our proposed network, YOLOV4-Lite + MobileNetV3 and YOLOV4-Lite +GhostNet detect all three objects, while the other network only detect two objects. The person(missed object) is too small and not obvious, so it is difficult to detect. The YOLOV4-Lite + our proposed network, YOLOV4-Lite + MobileNetV3 and YOLOV4-Lite +GhostNet detect all objects for two images. They have better detection performance than others.

To quantitatively analyze the performances of different networks, we use all images in the test dataset to test the detection performances of different networks. The performances for YOLOv4-Lite based on different convolution networks are shown in Table 10. YOLOv4-Lite based on our improved network has the highest mean average precision (mAP), followed by YOLOV4-Lite based on MobileNetV3. Due to that our improved network increases the new attention module in the MobileNetV2, so YOLOV4-Lite based on our improved network has more parameters and more detection time than YOLOV4-Lite based on MobileNetV2. However, the differences in parameters and detection between YOLOV4-Lite based on our improved network and YOLOV4-Lite based on other networks can be acceptable. This shows that our proposed network is more suitable for higher accuracy object detection.

**Table 10:** Results for different lightweight networks on object detection.

Lightweight network	mAP(%)	average detection time/image(ms)
YOLOV4-Lite+ShuffleNetV2	61.94	44.0
YOLOV4-Lite +GhostNet	64.64	65.8
YOLOV4 +HBONet	63.19	50.3
YOLOV4-Lite + MobileNetV3	64.86	46.2
YOLOV4-Lite + MobileNetV2	64.39	47.4
YOLOV4-Lite + our proposed network	67.28	49.5

## Conclusion

In this paper, we propose a lightweight neural network with higher accuracy based on MobileNetV2 and channel attention. Firstly, we analyze the influence of network depth on accuracy and propose reducing number of Bottleneck module of MobileNetV2 to realize balance between network depth, the network width and image resolution. Compared with original network, the accuracy of improved MobileNetV2 with modified network depth is increased by 0.66%, 0.52% and 0.55% on ImageNet-100 dataset, CIFAR-100 and CIFAR-10 dataset, respectively. Secondly, we introduce the channel attention mechanism into the Bottleneck module, which can make the network extract more effective features related to the object from complex background. Compared network without channel attention module, accuracy of the proposed network is increased by 2.15%. In the end, we also propose a new strategy to construct lightweight network by using the improved Bottleneck module and original Bottleneck module. Compared with other lightweight networks, our completed proposed method has better performance in accuracy for classification and object detection.

Although our proposed method has higher accuracy than other networks and is also a lightweight network, it consume more detection time than MobileNetV2. In our feature work, we will consider how to reduce the network complexity.

## References

[1] Kong L, Cheng J. Based on improved deep convolutional neural network model pneumonia image classification. PLOS ONE. 2020 Nov;16(11):e0258804. doi: 10.1371/journal.pone.0258804 PMC8568342 34735483

[2] Han H, Hao L, Cheng D, Xu H. GAN-SAE based fault diagnosis method for electrically driven feed pumps. PLOS ONE. 2020 Oct;15(10): e0239070. doi: 10.1371/journal.pone.0239070 33091004 PMC7581010

[3] Zhao L, Zhang Y, Cui Y. An Attention Encoder-Decoder Network Based on Generative Adversarial Network for Remote Sensing Image Dehazing. IEEE Sensors Journal. 2022 Jun;22(11):10890–10900. doi: 10.1109/JSEN.2022.3172132

[4] Junde C, Weirong C, Adnan Z, et al. Crop pest recognition using attention-embedded lightweight network under field conditions. Applied Entomology and Zoology. 2021 Mar;56:427–442. doi: 10.1007/s13355-021-00732-y

[5] Jiewen F, Xiaoyu T, Xingjiang J, Qunyuan Chen. Garbage Disposal of Complex Background Based on Deep Learning with Limited Hardware Resources. IEEE Sensors Journal. 2021 Sept; 21(8): 21050–21058.

[6] Khan S, Tufail M, Khan MT, Khan ZA, Iqbal J, et al. Real-time recognition of spraying area for UAV sprayers using a deep learning approach. PLOS ONE. 2021 Apr; 16(4): e0249436. doi: 10.1371/journal.pone.0249436 33793634 PMC8016340

[7] Hu X, Xu x, Xiao Y, et al. SINet: A Scale-insensitive Convolutional Neural Network for Fast Vehicle Detection. IEEE Transactions on Intelligent Transportation Systems. 2019 Mar; 20(3): 1010–1019. doi: 10.1109/TITS.2018.2838132

[8] Hu X, Xu x, Xiao Y, et al. Optimized MobileNet+ SSD: a real-time pedestrian detection on a low-end edge device. International Journal of Multimedia Information Retrieval. 2021 Nov; 10: 171–184.

[9] Zhang S, Wu Y, Men C, et al. Channel Compression Optimization Oriented Bus Passenger Object Detection. Mathematical Problems in Engineering. 2020 Mar; 2020: Article ID 3278235.

[10] Guo K, Hu B, Ma J, et al. Toward Anomaly Behavior Detection as an Edge Network Service Using a Dual-Task Interactive Guided Neural Network. IEEE Internet of Things Journal. 2021 Aug; 8(16):12623–12637. doi: 10.1109/JIOT.2020.3015987

[11] Zhao Z, Wu Q. Computer-Aided Recognition and Analysis of Abnormal Behavior in Video. Computer-Aided Design and Applications. 2020; 18(S3):34–45. doi: 10.14733/cadaps.2021.S3.34-45

[12] Nahshan Y, Chmiel B, Baskin C, et al. Loss aware post-training quantization. Machine Learning. 2021 Dec; 110(11):3245–3262. doi: 10.1007/s10994-021-06053-z

[13] Luo J H, Hao Z, Zhou H Y, et al. ThiNet: Pruning CNN Filters for a Thinner Net. IEEE Transactions on Pattern Analysis and Machine Intelligence. 2019 Oct; 41(10):2525–2538. doi: 10.1109/TPAMI.2018.2858232 30040622

[14] Wang J, Gou L, Zhang W, et al. DeepVID: Deep Visual Interpretation and Diagnosis for Image Classifiers via Knowledge Distillation. IEEE Transactions on Visualization and Computer Graphics. 2019 Jun;25(6):2168–1280. doi: 10.1109/TVCG.2019.2903943 30892211

[15] Howard AG, Zhu M, Chen B, et al. MobileNets: Efficient convolutional neural networks for mobile vision applications. 2017. [Online]. Available: https://arxiv.org/abs/1704.04861

[16] Howard AG, Zhu M, Chen B, et al. Mobilenetv2: Inverted residuals and linear bottlenecks. 2018 IEEE/CVF Conference on Computer Vision and Pattern Recognition (CVPR). 2018 Jun; 4510–4520.

[17] Howard A, Sandler M, Chen B, et al. Searching for MobileNetV3. IEEE International Conference on Computer Vision (ICCV). 2019 Nov; 1314–1324.

[18] Iandola FN, Han S, Moskewicz MW, et al. SqueezeNet: AlexNet-level accuracy with 50x fewer parameters and <0.5MB model size. 2016. [Online]. Available: https://arxiv.org/abs/1602.07360

[19] Gholami A, Kwon K, Wu B, et al. SqueezeNext: Hardware-aware neural network design. IEEE conference on computer vision and pattern recognition workshops (CVPRW). 2018 Jun; 1719–1730.

[20] Zhang X, Zhou X, Lin M, et al. ShuffleNet: An extremely efficient convolutional neural network for mobile devices. 2018 IEEE/CVF Conference on Computer Vision and Pattern Recognition (CVPR). 2018 Jun; 6848–6856.

[21] Ma N, Zhang X, Zheng H T, et al. ShuffleNet V2: Practical Guidelines for Efficient CNN Architecture Design. 2018. [Online].

[22] Han K, Wang Y, Tian Q, et al. GhostNet: More features from cheap operations. IEEE conference on computer vision and pattern recognition (CVPR). 2020 Jun; 1577–1586.

[23] Wang W, Li Y, Zou T, et al. A novel image classification approach via dense-MobileNet models. Mobile Information Systems. 2020 Jan; Article ID 7602384. doi: 10.1155/2020/7602384

[24] Li Y, Huang H, Xie Q, Yao L, Chen Q. Research on a surface defect detection algorithm based on MobileNet-SSD. Applied Sciences. 2018 Sep;8(9):1678. doi: 10.3390/app8091678

[25] Pan H, Pang Z, Wang Y, et al. A New Image Recognition and Classification Method Combining Transfer Learning Algorithm and MobileNet Model for Welding Defects. IEEE Access, 2020 Jun; 8:119951–119960. doi: 10.1109/ACCESS.2020.3005450

[26] Tan M, Le Q EfficientNet: Rethinking model scaling for convolutional neural networks. 2019. [Online]. Available: https://arxiv.org/abs/1905.11946

[27] Loshchilov I, Hutter F. Sgdr: Stochastic gradient descent with warm restarts. 2016. [Online]. Available: https://arxiv.org/abs/1608.03983.

[28] Li D, Zhou A, Yao A. HBONet: Harmonious bottleneck on two orthogonal dimensions. IEEE International Conference on Computer Vision (ICCV). 2019 Nov; 3315–3324.

[29] Bochkovskiy A, Wang C Y, Liao H Y M. Yolov4: Optimal speed and accuracy of object detection. [Online]. 2020. Available: http://arxiv.org/abs/1905.02423.

